# Mesenchymal stem cells for lung diseases: focus on immunomodulatory action

**DOI:** 10.1038/s41420-025-02303-4

**Published:** 2025-09-05

**Authors:** Yuqian Feng, Jiamin Lu, Jing Jiang, Kezhan Shen, Kaibo Guo, Yazhen Zhong, Shengyou Lin

**Affiliations:** 1 Department of Geriatrics, The First Affiliated Hospital of Zhejiang Chinese Medical University (Zhejiang Provincial Hospital of Traditional Chinese Medicine), Hangzhou, Zhejiang China; 2 Hangzhou School of Clinical Medicine, Zhejiang Chinese Medical University, Hangzhou, Zhejiang China; 3 The First School of Clinical Medicine, Zhejiang Chinese Medical University, Hangzhou, Zhejiang China; 4 Department of Oncology, The First Affiliated Hospital of Zhejiang Chinese Medical University (Zhejiang Provincial Hospital of Traditional Chinese Medicine), Hangzhou, Zhejiang China; 5 Department of Oncology, Affiliated Hangzhou First People’s Hospital, School Of Medicine, Westlake University, Hangzhou, Zhejiang China; 6 Department of Oncology, Hangzhou TCM Hospital of Zhejiang Chinese Medical University (Hangzhou Hospital of Traditional Chinese Medicine), Hangzhou, Zhejiang China

**Keywords:** Respiratory tract diseases, Stem-cell research

## Abstract

In recent years, the morbidity and mortality caused by acute and chronic lung diseases have gradually increased, becoming a global public health burden. However, modern medicine has yet to determine the exact treatment for lung diseases associated with inflammation. Alleviating lung diseases and repairing injured lung tissue are urgent issues that need to be resolved. Mesenchymal stem cells (MSCs) have been used to treat various inflammatory diseases owing to their powerful anti-inflammatory, anti-apoptotic, and tissue-regenerative properties. MSCs show great promise and have been shown to play a role in relieving lung diseases experimentally. The immune regulatory role of MSCs is thought to be a key mechanism underlying their multiple potential therapeutic effects. Immune cells and secreted factors contribute to tissue repair following lung injury. However, the overactivation of immune cells can aggravate lung injury. Here, we review evidence that MSCs act on immune cells to relieve lung diseases. Based on the immunomodulatory properties of MSCs, the specific mechanisms by which MSCs in alleviate lung diseases are reviewed, with a focus on innate and adaptive immunity. In addition, we discuss current challenges in the treatment of lung diseases using MSCs.

## Facts

- MSCs have a good application prospect in the treatment of lung diseases.
- MSCs can act on innate immune cells (neutrophils, macrophages, eosinophils) and adaptive
- immune cells (T cells, B cells) to play a repair role.
- The clinical application of MSCs still faces great challenges.

## Open Questions

- What is the specific mechanism by which MSCs regulate immune cells?
- Whether immune cells can affect the effect of MSCs?
- Can we develop strategies to enhance the activity of mesenchymal stem cells and overcome the challenges of clinical application?

## Introduction

Lung diseases, mainly caused by trauma, viral infections, air pollution, and aging populations, are the leading causes of death worldwide [1, 2]. In recent years, morbidity and mortality caused by acute and chronic lung diseases have gradually increased, becoming a global public health burden [3]. Chronic lung diseases, including chronic obstructive pulmonary disease (COPD), asthma, and idiopathic pulmonary fibrosis (IPF), affect more than 500 million people worldwide [4]. Acute lung injury (ALI) encompasses a wide range of pathological processes that can lead to severe acute respiratory distress syndrome (ARDS) with a mortality rate of up to 40% [5]. Both chronic and acute lung diseases are associated with inflammatory cell infiltration, pro-inflammatory cytokine secretion, alveolar epithelial and endothelial cell injury, and decreased alveolar fluid clearance [6, 7]. In the process of ALI, inflammatory cells such as monocytes and macrophages are activated to release pro-inflammatory factors (IL-6 and IFN-γ) to cause excessive pro-inflammatory reaction, while anti-inflammatory factors (IL-4 and IL-10) are secreted to jointly act on immune cells. The imbalance between pro-inflammatory and anti-inflammatory factors leads to the occurrence of ALI, which further develops into ARDS [8]. Neutrophil activation is an important part of the COPD process, which causes chronic mucous hypersecretion and destruction of lung substance through the release of neutrophil elastase and other bioactive substances [9]. Chronic airway inflammation is the main mechanism of asthma, and neutrophils, eosinophils and T lymphocytes are the main inflammatory cells involved [10]. Activated Th2 cells produce interleukin to activate B lymphocytes and synthesize specific IgE, which binds to IgE receptors on the surface of eosinophils. When the allergen re-enters the body, it binds to the IgE on the cell surface and produces a series of reactions. In addition, activated Th2 cell components of cytokines can also directly activate eosinophils and macrophages, causing them to gather in the airway [11]. In IPF patients, macrophages, neutrophils, and T cells increased significantly in alveolar lavage fluid, and the chemokines and cytokines released by them (such as TGF-β, IL-10, IL-4, IL-13) can promote fibrosis [12].

Modern medicine has thus far failed to pinpoint definitive therapies for inflammatory lung conditions, typically relying on anti-inflammatory agents to alleviate symptoms and reduce lung injury [13]. Presently employed medications, including non-steroidal anti-inflammatory drugs, corticosteroids, and bronchodilators, fail to impede the progression of the disease, while their associated side effects pose an additional challenge [14]. Mesenchymal stem cells (MSCs) have been used to treat various inflammatory diseases owing to their powerful anti-inflammatory, anti-apoptotic and tissue-regenerative properties. MSC-based therapies have become popular in regenerative medicine [15]. Thus, MSCs may be an ideal therapeutic agent for treating lung diseases. In animal models of lung injury, MSCs have been shown to have significant tissue repair effects [16]. These beneficial effects are mediated by multiple mechanisms, including reduced inflammation and permeability of alveolar epithelial and endothelial cells, enhanced alveolar fluid clearance, and reduced oxidative stress responses [15]. One of the reasons for the widespread study of MSCs is their powerful immunomodulatory properties, which play a role by suppressing innate and adaptive immune responses involving multiple immune cells [17]. A growing number of studies have found that MSCs can relieve lung diseases by regulating immune cells (such as neutrophils, macrophages, T cells, and B cells) [18–21].

This study aimed to summarize the specific mechanisms through which MSCs alleviate lung diseases by regulating immune cells based on their immunomodulatory properties. Therefore, we conducted a comprehensive literature search. In addition, we discuss the current challenges of MSC-based treatment of lung diseases, which will increase the possibility of applying this novel approach in the clinical treatment of lung diseases.

## MSCs on the innate immune responses during lung diseases

### Neutrophils

**Fig. 1:**
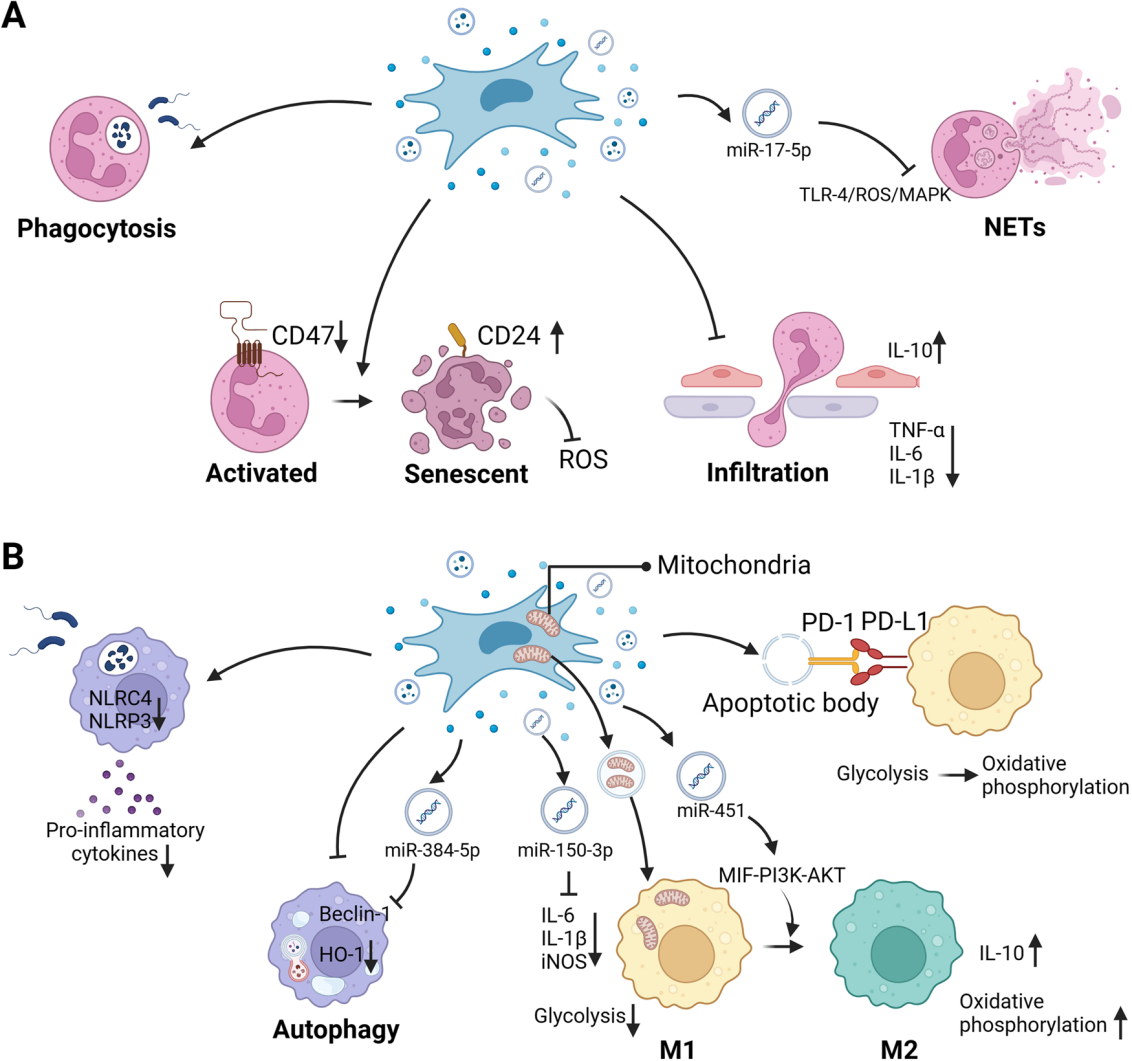
MSCs regulate innate immune cells. **A** MSCs regulate neutrophil. i) MSCs enhance phagocytosis activity; ii) MSCs convert activated neutrophils to senescent neutrophil phenotypes by upregating CD24 expression; iii) MSCs reduce neutrophil infiltration, decrease the expression of TNF-α, IL-6 and IL-1β, and increase IL-10; iv) MSCs derived exosomes can prevent excessive NETs formation by transferring miR-17-5p to target the TLR-4/ROS/MAPK pathway. **B** MSCs regulate macrophage. i) MSCs inhibit the activation of NLRP3 and NLRC4 inflammasome in macrophages and improved phagocytosis function, thereby inhibiting inflammation; ii) MSCs inhibit macrophage autophagy through miR-384-5p/Beclin-1 and HO-1 signaling pathways; iii) MSC-Exo miR-150-3p inhibits M1 polarization by down-regulating IL-6, IL-1β, iNOS, and promotes M2 polarization by up-regulating IL-10 in LPS-stimulated macrophages; iv) MSC-Exos can transfer stem cell-derived mitochondrial components to alveolar macrophages; v) MSC-Exo miR-451 regulates the MIF-PI3K-AKT signaling pathway to promote the polarization of macrophages M1 to M2; vi) PD-1 expressed on apoptotic bodies interacts with PD-L1 expressed by macrophages to shift macrophage metabolism from glycolysis to oxidative phosphorylation.

The activation and recruitment of neutrophils play important pathological roles in ALI. During acute inflammatory responses, neutrophils rapidly recruit inflamed tissues from the bloodstream via a tightly controlled multi-step recruitment cascade, and are the first white blood cells to reach a site of infection or injury [22]. Activated neutrophils control injured lesions and remove cell debris [23]. Although neutrophil activation is critical for host defense, overactivation releases a variety of toxic substances, including reactive oxygen species (ROS), pro-inflammatory cytokines (such as nuclear factor-κB (NF-κB), Interleukin (IL)-1β, and IL-17), and proteases [24]. Toxic substances released by neutrophils trigger various chemotactic signals that enhance inflammatory responses [23]. Considering the important role of neutrophils in the pathogenesis of ALI, neutrophils targeting is a new approach for ALI treatment. Several studies have shown that MSCs and extracellular vesicles (EVs) inhibit neutrophil migration and infiltration, reduce neutrophil mediated oxidative stress, and release inflammatory factors that may have protective effects on lung injury [25] (Table 1, Fig. 1A).

**Table 1:** The ways MSCs on the innate immune responses during LI.

Lung Diseases	Animal Model	Intervention (MSCs/EVs)	Regulation of Immune Cell	Outcomes	Mechanism	References
ALI	Injected intratracheal with PA in C57BL/6 mice	AMSCs	Increased the phagocytic ability of macrophages; Reduced white blood cell count	Reduced the bacterial load, inflammation of lung tissue and histopathological damage	Reduced the activation of NLRC4 inflammasome	[97]
ALI	Injected intraperitoneally with LPS in C57BL/6 mice	AMSC-CM activated by flagellin	Induced macrophage polarization to M2 profile	Alleviated the lung exudation; Inhibited inflammatory cell recruitment in lung tissue; Decreased the concentration of inflammatory factors	/	[98]
ALI	Injected intraperitoneally with LPS in BALB/c mice	BMSCs	Induced further macrophage polarization to M2 profile; Decreased absolute numbers of neutrophils	Inibited Inflammatory and oxidative stress reaction	Activated STC2/Nrf2 pathway	[31]
ALI	Injected intraperitoneally with CEES in C57BL/6 mice	AMSCs	Prevented the differentiation of CEES-stimulated macrophages into M1 phenotype and stimulated the polarization to M2 phenotype	Reduced progressive histopathologic changes in the lung; Reduced inflammatory cytokines	/	[99]
ALI	Injected intraperitoneally with LPS in Sprague-Dawley rats	pMSCs	Reduced the expression of TNF-α and increased IL-10 in RAW264.7 macrophage inflammation model; Reduced white blood cell count	Alleviated the infiltration of inflammatory cells, pulmonary hyperemia and hemorrhage, and interstitial edema	Downregulated CXCL12	[100]
ALI	Injected intraperitoneally with LPS in Sprague-Dawley rats	BMSC-Exos	Prevented LPS-induced alveolar macrophage apoptosis and autophagy stress	Improved pathological changes in lung tissue; Improved pulmonary vascular permeability; Regulated the inflammatory cytokines	Regulated miR-384-5p/Beclin-1 pathway	[101]
ALI	Injected intraperitoneally with LPS in C57BL/6 mice	HS-pretreatedhUCMSCs	Enhanced immunoregulatory effect in inducing M2 macrophage polarization; Decreased absolute numbers of neutrophils	Improved the pathological changes and lung damage-related indexes; Reduced the proinflammatory cytokine levels	Inhibited NLRP3 inflammasome activation	[102]
ALI	/	BMSCs	Down-regulated the elevated levels of autophagy in macrophages	/	Activated PI3K/Akt/ HO-1 signaling pathway	[39]
ALI	Injected intraperitoneally with LPS in C57BL/6 mice	MSC-Exos	Inhibited LPS-induced glycolysis in macrophages and production of proinflammatory cytokines	Alleviated sepsis-induced ALI and systemic inflammation; Improved survival rate	/	[103]
ALI	Severe burn Sprague-Dawley rats	hUCMSC-Exos	Modulated macrophage M2 polarization	Reduced inflammation and oxidative stress	Regulated miR-451 /MIF/PI3K/AKT signaling pathway	[43]
ALI	Injected intraperitoneally with LPS in C57BL/6 mice	hUCMSCs	Increased PD-L1 expression in the lung macrophages	Decreased total protein exudation concentration and cell number in BALF; Reduced pathological damage and inflammation	Regulated COX2/PGE2 signaling pathway	[36]
ALI	Injected with LPS in C57BL/6 mice	BMSCs	Suppressed the activation of alveolar macrophages	Decreased total protein exudation concentration; Alleviated alveolar epithelial damage; Reduced inflammation	Regulated PGE2/EP4R signaling pathway	[104]
ALI	Ligated and punctured cecum in C57BL/6 mice	AMSC-Exos	Inhibited the LPS-mediated release of IL-27 in macrophages; Reduced the number of pulmonary macrophages	Decreased pulmonary edema and pulmonary vascular leakage; Reduced inflammation	/	[105]
ALI	Injected with LPS in C57BL/6 mice	AMSC-Exos	Rendered macrophages shifting from M1 proinflammatory to M2-polarized anti-inflammatory phenotype	Alleviated lung inflammation and injury	Transferred mitochondrial component	[46]
ALI	Injected with LPS in C57BL/6 mice	Nrf2 -hAMSC-EVs	Promoted M2-like polarization; Inhibited infiltration of neutrophils	Reduced apoptosis and inflammation	Inhibited the activation of the NLRP3 inflammasome	[106]
ALI	Injected with LPS in C57BL/6 mice	Hypoxia-preconditioned MSC-CM	Promoted anti-inflammatory polarization; Restored efferocytosis of macrophages	Promoted resolution of inflammation	/	[107]
ALI	Injected with LPS in C57BL/6 mice	MSC-EVs	Promoted M2-like polarization	Reduced the expression of pro-inflammatory cytokines, increased the expression of anti-inflammatory cytokines; Decreased pathological scores	/	[108]
ALI	Ligated and punctured cecum in mice	AMSC-Exos	Promoted M2 polarization and TGF -β secretion	Inhibited inflammatory responses	/	[109]
ALI	Injected with LPS in C57BL/6 mice	PEG2-MSCs	Promoted M2 polarization; Decreased absolute numbers of neutrophils	Reduced cellular infiltration; Reduced histopathological changes and pro-inflammatory cytokines and increased anti-inflammatory cytokines	/	[32]
ALI	Injected with LPS in C57BL/6 mice	UCMSC-ABs	Inhibited pro‐inflammatory polarization and cytokines production of macrophages; Decreased absolute numbers of neutrophils	Suppressed lung inflammation	Regulated PDL1–PD1 pathway; Reprogrammed metabolic pathways	[110]
ALI	Injected with *E.coil* in ICR mice	UCMSCs	Regulated on M1/M2 macrophage polarization; Decreased absolute numbers of neutrophils	Attenuated lung injury and inflammation	Secreted SOCS3	[111]
ALI	Cardiopulmonary bypass related lung injury in Sprague-Dawley rats	BMSC-Exos	Suppressed ROS production and down-regulated the levels of inflammatory cytokines of macrophages	Attenuated histological changes; Down-regulated inflammatory cytokine levels; Alleviated oxidative stress	Regulated NF-κB p65 and Akt/Nrf2/HO-1 signaling pathways	[112]
ALI	/	LPS-BMSC-Exos	Suppressed pro-inflammatory polarization and promoted anti-inflammatory polarization of alveolar macrophages	Suppressed lung inflammation	Regulated miR- 150-3p/ INHBA signaling pathway	[44]
ARDS	Injected intraperitoneally with LPS in C57BL/6 mice	BMSC-Exos	Ameliorated LPS-induced alveolar macrophage M1 polarization	Exerted anti-inflammatory effects	Inhibited glycolysis via inhibition of HIF-1α	[113]
ARDS	Injected with LPS in BALB/c mice	hUCMSCs	Promoted M2-like polarization; Decreased absolute numbers of neutrophils	Improved lung injury; Attenuated inflammatory cell infiltration	/	[114]
ARDS	Injected with *Klebsiella pneumoniae* in C57BL/6 mice	PMSCs	Preserved resident alveolar macrophage over bone marrow–recruited macrophage and drived the overall milieu to an M2 immunomodulatory phenotype; Enhanced multiple antibacterial functions in alveolar macrophage	Decreased pulmonary inflammation and tissue injury	Secreted IL-1β	[115]
ARDS	/	hUCMSCs	Increased IL-10 expression in alveolar macrophages; Induced alveolar macrophage polarization	/	Regulated STC1/ PI3K/AKT/mTOR pathway	[37]
Asthma	Injected intraperitoneally with OVA in BALB/c mice	hUCMSCs	Reduced M2 macrophages; Decreased absolute numbers of eosinophils	Reducted airway hyperresponsiveness and inflammation	/	[52]
Asthma	Instillated intranasally with total house dust mite extracts in C57BL/6 mice	Serum from asthmatic mice-stimulated BMSCs	Induced further macrophage polarization to M2 profile; Decreased absolute numbers of neutrophils and eosinophils	Reducted lung inflammation and remodeling; Improved lung mechanics	/	[53]
Asthma	Injected intraperitoneally with OVA/CFA in BALB/c mice	hUCMSC-Exos	Regulated macrophage polarization; Decreased absolute numbers of neutrophils	Reduced inflammation	Regulated NF-κB and PI3K/AKT signaling dependent on TRAF1	[116]
Asthma	Injected intraperitoneally with OVA in BALB/c mice	MSC-EVs	Inhibited the recruitment and polarization of lung macrophages; Decreased absolute numbers of eosinophils	Ameliorated allergic airway inflammation	/	[54]
Asthma	Injected intraperitoneally with OVA in C57BL/6 mice	MSC-Exos	Enhanced lung interstitial macrophages ratios and high level of IL-10; Decreased absolute numbers of eosinophils	Decrease inflammation index, histological mucus index, total cells and cytokines	/	[55]
Asthma	IL-13 transgenic mice	Liproxstatin-1-primed hUCMSCs/ hUCMSCs	Alterated lung macrophage populations; Decreased absolute numbers of neutrophils and eosinophils	Reduced airway inflammation and fibrosis	/	[117, 118]
Asthma	Injected intraperitoneally with IL-33 in C57BL/6 mice	iPSC-MSC-EV	Mitigated the activation of ILC2s	Ameliorated ILC2-dominant allergic airway inflammation	Delivered miR-146a-5p	[56]
Asthma	Instillated intranasally with total house dust mite extracts in BALB/c mice	BMSCs	Decreased absolute numbers of neutrophils, eosinophils and lymphocytes; Induced lung macrophage polarization into suppressive phenotype	Inhibited airway hyper-responsiveness and bronchoconstriction; Decreased airway inflammation	/	[119]
Asthma	Instillated intranasally with total house dust mite extracts in C57BL/6 mice	Eicosapentaenoic acid -stimulated BMSCs	Induced macrophage polarization to the M2; Decreased absolute numbers of neutrophils and eosinophils	Reduced lung morphological changes, remodeling, and mucus hypersecretion; Improved lung mechanics	/	[120]
IPF	Injected intratracheal with bleomycin in C57BL/6 mice	hUCMSCs	Reduced M2c subset of M2 monocyte-derived macrophages; Decreased absolute numbers of neutrophils and eosinophils	Attenuated inflammation and the degree of lung fibrosis	/	[121]
IPF	Injected intratracheal with bleomycin in C57BL/6 mice	hAMSCs	Increased macrophage polarization toward M2, and reduced the antigen-presentation potential of macrophages and dendritic cells	Decreased alveolar obliteration; Decreased extracellular matrix proteins and α‐SMA	/	[21]
BPD	Hyperoxia-induced in FVB mice/ Sprague-Dawley rats	MSC-Exos	Regulated macrophage phenotype	Restored lung architecture; Improved pulmonary development and ameliorates septal fibrosis; Rescued loss of peripheral pulmonary blood vessels and peripheral pulmonary arterial remodeling; Modulated inflammation	/	[122, 123]
BPD	Hyperoxia-induced in pregnant Sprague-Dawley rats	hUCMSCs	Regulated Macrophage Polarization	Attenuated inflammation	Regulated PTX3/TSG14 pathway	[124]
BPD	Hyperoxia-induced in mice	hUCMSCs	Decreased FPR2 levels in alveolar macrophages	Reduced levels of inflammatory cytokines (IL-1α and TNF-α)	/	[125]
Silicosis	Instilled intratracheally silica suspension in C57BL/6 mice	BMSCs	Decreased the macrophage infiltration	Attenuated inflammation	Regulated the activation of inflammasome by secreting TSG-6	[126]
PAH	Injected with the VEGF receptor 2 antagonist in Sprague-Dawley rats	MSC-EVs	Induced further macrophage polarization to M2 profile	Reduced right ventricular hypertrophy and muscularization of peripheral pulmonary vessel	/	[127]
DAH	Injected intraperitoneally with sterile filtrated pristine in C57BL/6 J mice	hUCMSC-Exos	Enhanced M2 polarization	Alleviated pathological symptoms; Attenuated the alveolar injuries and inflammatory responses	/	[128]
RILI	Radiated with 20 Gy 60Co γ-ray in Sprague-Dawley rats	miR-21-knockout BMSCs	Controlled macrophage polarization	Decreased RILI-induced acute inflammation; Reduced mortality in rats with RILI	/	[129]

*iPSCs* Induced pluripotent stem cells, *ILC2s* Group 2 innate lymphoid cells, *BPD* Bronchopulmonary dysplasia, *Exos* Exosomes, *HIF-1α* Hypoxia-inducible factor 1α, *PTX3* Pentraxin 3, *TSG14* Tumor necrosis factor-inducible gene 14, *PAH* Pulmonary arterial hypertension, *VEGF* vascular endothelial growth factor, *STC2* Stanniocalcin-2, *Nrf2* Nuclear factor erythroid 2-related factor 2, *CEES* 2-Chloroethyl ethyl sulfide, *pMSCs* Placenta-derived mesenchymal stem cells, *DAH* Diffuse alveolar hemorrhage, *HS* Heat shock, *NLRP3* NLR family pyrin domain containing 3, *PI3K/Akt* Phosphoinositide 3-kinase/protein kinase B, *HO-1* Heme oxygenase-1, *PD-L1* programmed cell death protein ligand 1, *COX2* Cyclooxygenase-2, *PEG2* Prostaglandin E2, *ABs* Apoptotic bodies, *SOCS3* suppressor of cytokine signaling 3, *INHBA* Inhibin subunit beta A, *TSG6* Tumor necrosis factor-stimulated gene 6, *STC1* Stanniocalcin-1, *NF-κB* nuclear factor-κB

Once neutrophils are over-activated, ROS may exceed the cell’s clearance capacity and are released into the extracellular environment in large quantities, causing harm to the lung tissue [26]. ROS can act as both a messenger of tumor necrosis factor (TNF)-induced cell death and a regulator of inflammation-related signaling pathways, such as c-Jun N-terminal kinase (JNK) and NF-κB [27]. A series of studies have demonstrated the anti-oxidative stress effect of MSCs. MSC therapy converts activated neutrophils into senescent neutrophils by upregulating CD24 expression, thereby inhibiting inflammation by reducing ROS production, and nicotinamide adenine dinucleotide phosphate oxidase [28]. Moreover, in a bleomycin-induced PF model, gingival-derived MSCs intervention significantly down-regulated MDA and MPO levels, up-regulated GSH and SOD levels, and alleviated oxidative stress in lung tissue [29]. ILs released by overactivated neutrophils have a variety of functions in inflammation, are associated with the progression of ALI [30]. MSCs and EVs have been shown to reduce the infiltration of neutrophils and proinflammatory cytokines (such as IL-1β, IL-17, TNF-α, and IL-6), while increasing the expression of anti-inflammatory cytokines (such as IL-10) in injured lung tissue [31, 32]. Furthermore, when neutrophils are exposed to large numbers of bacteria and fungi, extracellular DNA and histone, as well as cytoplasmic proteases, antimicrobial peptides and oxidant molecules form neutrophil extracellular traps (NETs). NETs can intensify the inflammatory response during lung injury and promote macrophage polarization to the M1 phenotype [33]. MSCs is a promising NET targeted therapy. Soluble factors secreted by MSCs effectively inhibit NET production, thereby alleviating inflammation [34]. In addition, Chu *et al*. found that hypoxic-pretreated MSC-derived exosomes could prevent excessive NETs formation by transferring miR-17-5p to target the TLR-4/ROS/MAPK pathway, thereby speeding up wound healing [35]. It can be seen that MSCs affect neutrophils in multiple ways, thereby alleviating various lung diseases.

### Macrophages

Macrophages in the lung tissues play a central role in inflammatory responses. Several preclinical studies have shown that MSCs and their secretory factors can repair lung tissue damage by targeting macrophages (Table 1, Fig. 1B). MSCs and their EVs can reduce the infiltration of macrophages, lower the levels of pro-inflammatory cytokines in macrophages, increase the levels of anti-inflammatory factors, as well as improve their phagocytic function, ultimately improve the lung tissue damage [36, 37]. Additionally, macrophage autophagy is closely associated with various lung diseases. Moderate autophagy is thought to protect cells from hypoxia and starvation, whereas overactivated autophagy can lead to apoptosis or necrosis [38]. However, Bone marrow-derived MSCs (BMSCs) and exosomes regulate autophagy in macrophages through phosphoinositide 3 kinase (PI3K)/ Protein Kinase B (Akt)/ heme oxygenase 1 (HO-1) pathway and by delivering miR-384-5p [39].

Under diverse environmental conditions, macrophages can polarize into distinct phenotypes, including classically activated M1 and selectively activated M2 macrophages [40]. When stimulated by LPS or Th1-associated cytokines, such as IFN-γ and TNF-α, macrophages can be polarized into an M1 phenotype. M1 macrophages exhibit heightened production of proinflammatory cytokines, leading to tissue injury, while concurrently facilitating host immune clearance of pathogens. M2 macrophages are usually induced by IL-4, IL-13, TGF-β, and M-CSF, which mainly secrete anti-inflammatory cytokines that promote wound healing and tissue damage repair [41]. Through the maintenance of immune homeostasis within the lung microenvironment, both M1 and M2 macrophages demonstrate the capacity to avert excessive inflammatory responses that precipitate tissue injury [42]. Thus, maintaining the balance between M1 and M2 macrophages is a promising strategy for treating lung injury. MSCs can regulate M1/M2 polarization of macrophages through a variety of specific mechanisms and play an important role in lung injury. Lv et al. found that MSCs mediate macrophage polarization by regulating Stanniocalcin-2, a stress- response protein with antioxidant properties, thereby alleviating lung inflammation and oxidative stress in ALI mice [31]. In addition, MSC-derived exosomes (MSC-Exos) regulate the downstream MIF-PI3K-AKT signaling pathway and inflammatory mediators (down-regulate IL-6, IL-1β; up-regulate IL-10) by delivering different non-coding RNAs (miR-451 and miR-150-3p), thus promoting the polarization of M1 to M2 macrophages [43, 44]. Mitochondria produce energy to support cellular activities, such as cell proliferation, apoptosis, and metabolism. Dysfunctional mitochondria can disrupt the metabolic health of alveolar epithelial cells and macrophages, leading to various lung diseases [45]. It is worth noting that adipose derived mesenchymal stem cell (AdMSC)-Exos can transfer stem cell-derived mitochondrial components to alveolar macrophages, improve the mitochondrial integrity of macrophages, transform macrophages into anti-inflammatory phenotypes, restore immune homeostasis, and thus relieve lung inflammation [46]. Due to the presence of human microenvironment, injected MSCs undergo programmed apoptosis and release apoptotic vesicles. Apoptotic MSCs exhibit distinctive anti-inflammatory effects and exert immunomodulatory effects [47]. Compared to normal human umbilical cord MSCs (hUC-MSCs), apoptotic hUC-MSCs can more effectively reduce inflammatory exudates and vascular permeability in the lungs of ALI rats [48]. Using a mouse model of ALI, Jiang et al. demonstrated that apoptotic bodies released by transplanted hUC-MSCs transformed macrophages from a pro-inflammatory to an anti-inflammatory state. The specific mechanism is that PD-L1 expressed by apoptotic bodies interacts with PD-1 on macrophages, which changes the metabolism of macrophages from glycolysis to oxidative phosphorylation [49]. Recently, nanotechnology utilizing the complete natural cell membrane coating of MSCs has been an emerging platform for targeted therapies. Lu et al. successfully constructed a novel nanoparticle drug carrier system for sepsis management by modifying nanoparticles with LPS-treated BMSC membranes and delivering them to the infectious microenvironment with a silver metal-organic framework as the nanocore, which exerts both anti-inflammatory and antibacterial effects, alleviates cytokine storms, and protects vital organ functions [50].

### Eosinophils

Asthma is a chronic inflammatory airway ailment in which eosinophils play a significant role. Eosinophils are end-effector cells involved in allergic diseases. Following the receipt of stimulus signals, eosinophils perform immunomodulatory and proinflammatory functions by releasing various immunomodulatory factors, such as cytokines, chemokines, growth factors, and cytotoxic proteins [51]. Multiple studies have shown that MSCs and their EVs can reduce the number of eosinophils in the lung tissue of asthmatic mice, thereby reducing allergic airway inflammation and remodeling [52–55] (Table 1). Moreover, Group 2 innate lymphoid cells (ILC2) mediate the activation of eosinophils in the airway during asthma, and ILC2 is associated with persistent pulmonary eosinophilia. Small EVs derived from human mesenchymal cells inhibit ILC2 levels, inflammatory cell infiltration and airway hyperreactivity in asthmatic mice by delivering miR-146a-5p [56]. Therefore, MSCs can relieve asthma by regulating eosinophils and ILC2.

## MSCs on the adaptive immune responses during lung diseases

### T cells

CD4 + T cells are the main cells involved in the adaptive immune response and play an important role in body development and homeostasis. Under the synergistic effect of T cell receptor stimulation and cytokines, naïve CD4 + T cells differentiate into distinct subpopulations, including Th1, Th2, Th17, and Regulatory T (Tregs) cells [57]. Tregs secrete anti-inflammatory factors (such as TGF-β), promote neutrophil apoptosis, diminish neutrophil numbers, and foster a conducive environment for tissue repair [58]. In addition, Tregs regenerate the alveolar epithelium and induce the proliferation and differentiation of type II alveolar cells into type I cells [59]. Th17 cells produce IL -17, which induces the secretion of pro-inflammatory factors. IL-17 exerts a direct influence on monocytes by facilitating their maturation and extravasation, thereby inducing the recruitment of macrophages [60]. IL-17 also contributes to the generation of oxidizing free radicals, which can exacerbate damage to alveolar epithelial and microvascular endothelial cells [61]. Therefore, the Th17/Treg balance is critical for the progression of lung injury. A ratio of Th17 cells to Tregs greater than 0.79 was an independent predictor of poor prognosis patients [62]. Lung-resident MSCs prevent the differentiation of naïve CD4 + T cells into Th17 cells in vitro, inhibit the production of IL-17 and IL-22 by fully differentiated Th17 cells, and induce a Treg phenotype [63]. Similarly, MSCs transplantation in animals can significantly increase the levels of IL-10, Foxp3 and Tregs in peripheral blood and lung tissue samples, down-regulate the levels of IL-17 and Th17, regulate the balance of Treg/Th17, and ultimately alleviate lung diseases [64, 65].

**Fig. 2:**
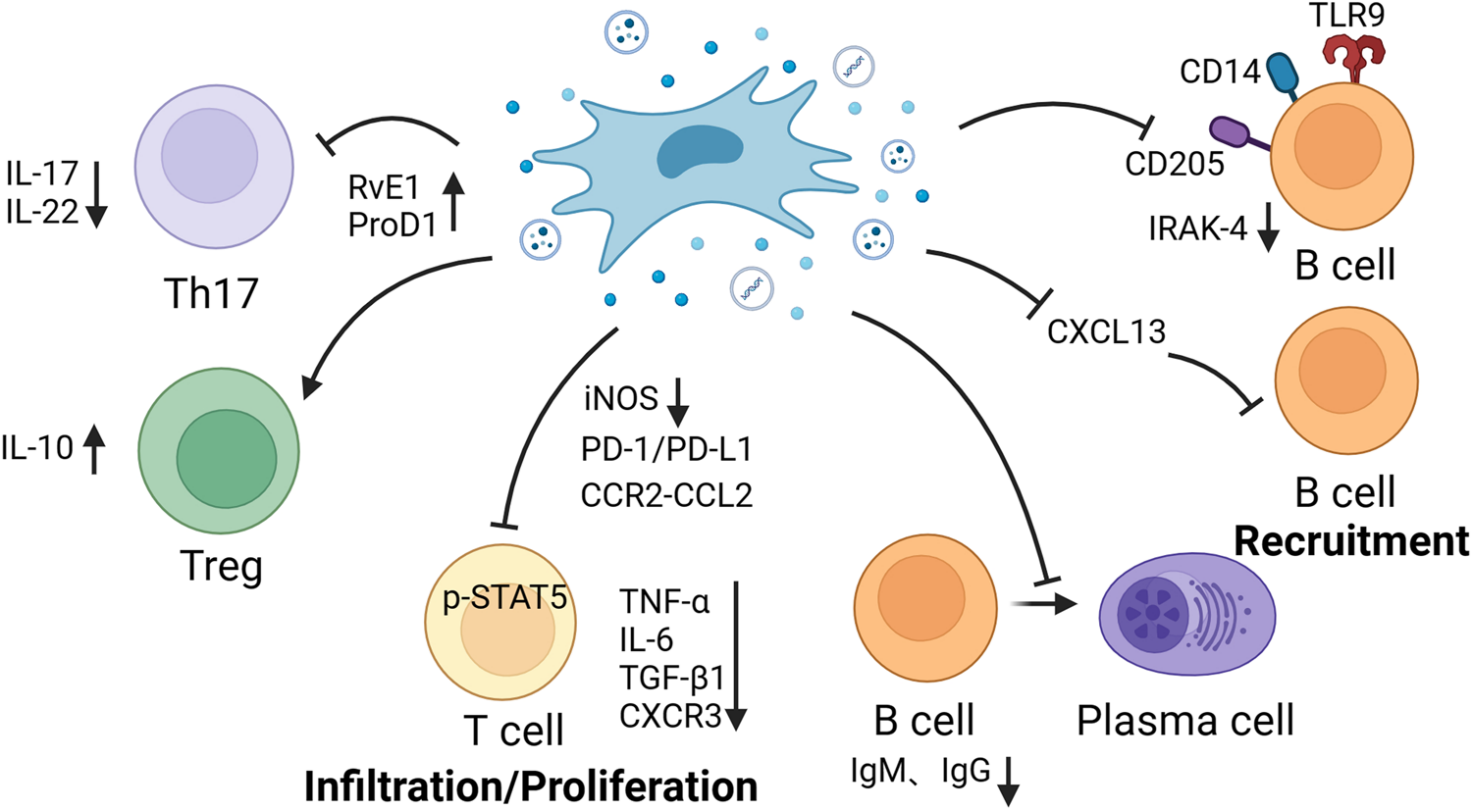
MSCs regulate adaptive immune cells. i) MSCs inhibit the production of IL-17 and IL-22 by fully differentiated Th17 cells, and increase IL-10 by induced the Tregs phenotype; MSCs promote proresolving mediators protectin D1, resolvin E1 and regulating Treg/Th17 balance; ii) MSCs alter the CCR2-CCL2 and PD-1/PD-L1 axis, reducing T cell infiltration; MSCs inhibit the proliferation of T cells, which is related to the reduction of iNOS expression in MSCs and the phosphorylation of STAT5 in T cells; iii) MSCs inhibit the proliferation and differentiation of B cells into plasma cells and the production of IgM and IgG; iv) MSCs influence the dynamics of B-cell recruitment by inhibiting the chemokine CXCL13; v) MSCs reduce the expression of CD205, TLR9, and CD14 and IRAK-4 expression.

Following lung injury, immune cells and secreted cytokines form an inflammatory microenvironment that promotes fibrosis [66]. Tu’s team have found that human CD8 + T cells are essential for the induction of PF in mice. MSCs can alleviate PF and improve lung function by inhibiting bleomycin-induced CD8 + T cell invasion and proinflammatory cytokine production, which are related to the regulation of programmed death-1/programmed death-ligand 1 pathway [20]. After MSC treatment, CD8 + T cells in the lungs expressed low levels of CXCR3. MSCs may reduce lung injury caused by neutrophil infiltration by inhibiting CD8 + T cell chemotaxis [67]. In addition to affecting CD8 + T cells, MSCs also reduce inflammatory CD3 + T cell infiltration, expression of inflammatory cytokines TNF-α, IL-6, TGF-β1, and lactate level to improve PF induced by paraquat [68] (Table 2, Fig. 2).

**Table 2:** The ways MSCs on the adaptive immune responses during LI.

Lung diseases	Animal model	Intervention (MSCs/EVs)	Regulation of immune cells	Outcomes	Mechanism	References
IPF	Aspirated bleomycin in C57BL/6 mice	BMSCs	Increased numbers of Tregs	Reduced fibrosis and inflammation; Up-regulated circulating TGF-β1	/	[130]
IPF	Instilled intratracheally with bleomycin in C57BL/6 mice	hAMSCs	Increased Treg cell marker Foxp3; Reduced pulmonary B-cell recruitment, retention, and maturation	Decreased alveolar obliteration; Decreased extracellular matrix proteins and α‐SMA	/	[21]
PF	Injected intratracheally with bleomycin in Rag2^−/−^γc^−/−^ mice	BMSCs	Inhibited the proliferation of anti-CD3–stimulated human CD45 lymphocytes as well as CD4 and CD8 T cells	Suppressed proinflammatory cytokines such as IFN-γ, TNF-α, and IP-10	PD-1/PD-L1 Pathway	[20]
PF	Administered paraquat to Sprague-Dawley rats	hAMSCs	Reduced CD3 + T lymphocyte accumulation	Decreased collagen deposition; Improved histopathological changes; Decreased levels of lactic acid and inflammatory cytokines in the plasma	/	[68]
ALI	Injected intratracheally with LPS in C57BL/6 mice	MSCs	Decreased immunoglobulin-related gene expression in lung B cells	Decreased chemokines CCL3, CCL4 and IL-6, IFN-γ	/	[75]
ALI	Injected intratracheally with LPS in Balb/C mice	hUCMSCs	Increased CD4 + CD25+ Foxp3+Treg cells; Decreased IFN-r-producing Th1 cells	Reduced inflammatory cytokines; Increased anti- inflammatory factor IL-10	/	[131]
ALI	Injected intratracheally with LPS in C57BL/6 mice	LRMSCs	Increased the percentage of Tregs and reduced the percentage of Th17 cells	Reduced inflammatory cells and decreased the inflammatory cytokines IL-1β, IL-6, and TNF-α; Increased the expression of KGF-2 and SPC; Decreased deposition of interstitial collagen; Increased IL-10 and reduced IL-17, IL-22	/	[63]
ALI	Injected intratracheally with LPS in C57BL/6 mice	BMSCs	Increased the percentage of Treg cells and decreased the percentage of Th17 cells	Decreased MPO activity; Attenuated IL-6 and IL-17	/	[64]
ALI	Injected intratracheally with LPS in C57BL/6 mice	MSCs	Down-regulated Ly6C^+^CD8^+^T cells	Reduced inflammatory factors	/	[67]
Asthma	Injected intraperitoneally with OVA in BALB/c mice	BMSCs	Increased the frequencies of CD4 + CD25+Treg cells	Reduced the number of inflammatory cells and airway hyperresponsivensess; Increased IL-10 and IL-12	Regulated Jagged-1 signalling	[132, 133]
Asthma	Injected intraperitoneally with OVA in BALB/c mice	AMSCs/ hUCMSCs	Enhanced Tregs expansion	Reduced airway hyperresponsivensess, lung inflammation, and mucus production; Decreased IgE and IgG1; Down-regulated Th2 cytokines and up-regulated Th1 cytokines	/	[134, 135]
Asthma	Injected intraperitoneally with OVA in Sprague-Dawley rats	hPMSCs	Increased the frequencies of Treg cells; Reduced the frequencies of Th17 positive cells	Reduced the number of inflammatory cells; Increased IL-10 and reduced IL-17	/	[65]
Asthma	Injected intraperitoneally with OVA in BALB/c mice	IFN -γ-and ‘ IFN -γ + TNF -α‘ treated AMSCs	Improved Treg cell and Th9 related parameters ratio and shift T cell response toward Treg cells	Attenuated lung inflammation; Reduced IL-9 and increased IL-35	/	[136]
Asthma	Inoculate with *Aspergillus fumigatus* hyphal extract in C57Bl/6 mice	BMSCs/ BMSC-CM/BMSC-EVs	/	Alleviated airway hyper-responsiveness and inflammation; Decreased of Th2 and Th17 soluble mediators	/	[137, 138]
Asthma	Instillated intranasally with house dust mite in BALB/c mice	BMSCs	Reduced the number of activated and antigen-acquiring CD11c + CD11b+ dendritic cells	Alleviated airway hyper-responsiveness and inflammation; Decreased the expression of the pro-Th2 cytokine IL-25	/	[139]
Asthma	Injected intraperitoneally with OVA in BALB/c mice	hUCMSCs	/	Alleviated lung inflammation; Reduced Th2 cytokine IL-5	/	[140]
Asthma	Injected intraperitoneally with OVA in Wistar rats	BMSC-CM	/	Modulated the T-bet, GATA-3, IL-4 and IFN-γ	/	[141]
Asthma	Instillated intranasally with *Alternaria alternata* and house dust mite /diesel exhaust particle in BALB/c mice	hUCMSCs	/	Down-regulated the IL-5 and IL-13 production of differentiated mouse Th2 cells and peripheral blood mononuclear cells	/	[142]
COPD	Sprague-Dawley rats were exposed to tar and nicotine	BMSCs	Reduced the frequency of Th17 and Th1 cells, increased the frequency of Tregs	Mitigated anti-elastin responses and inflammatory infiltrates; Modulated the imbalance of cytokine and chemokines	/	[143]
COPD	Sprague-Dawley rats were exposed to nicotine	BMSCs	inhibited the proliferation of nicotine‑exposed T cells	/	Down-regulated iNOS expression in BMSCs and enhanced STAT5 phosphorylation in T cells	[144]
LCHS	Used the blast wave of a percussive nail gun in Sprague-Dawley rats; Withdrawn blood then re-infused in Sprague-Dawley rats	MSCs	Expanded the Treg cell population	Decreased total lung injury score	/	[145]
RILI	Radiated with 20 Gy ^60^Co γ-ray in C57BL/c mice	Decorin-modified UCMSCs	Down-regulated Treg cells	Attenuated histopathological injury; Inhibited inflammatory cytokines and Chemokines; Alleviated lung fibrosis	/	[146]
IPS	Radiated with 8 Gy 60Co γ-ray in BALB/c mice	BMSCs	Inhibited T cell activation and proliferation	Alleviated lung pathological damage	Regulated CCR2-CCL2 axis	[147]

*IPF* Idiopathic pulmonary fibrosis, *ALI* Acute lung injury, *LCHS* Lung contusion followed by hemorrhagic shock, *PF* Pulmonary Fibrosis, *COPD* Chronic obstructive pulmonary disease, *IPS* Idiopathic pneumonia syndrome, *hAMSCs* Human amniotic membrane mesenchymal stromal cells, *CM* Conditioned medium, *hUCMSCs* Human umbilical cord mesenchymal stem cells, *BMSCs* Bone marrow-derived mesenchymal stem cells, *AMSCs* Mesenchymal stem cells derived from adipose tissue, *hPMSCs* human placenta mesenchymal stem cells, *LRMSCs* Lung-resident- mesenchymal stem cells, *EVs* Extracellular vesicles, *Treg cell* T regulatory cell, *IL-6* Interleukin-6, *IFN-γ* Interferon-γ, *KGF* Keratinocyte growth factor, *SPC* surfactant protein C, *LPS* Lipopolysaccharide

### B cells

In conjunction with T cells, B cells are vital components of the adaptive immune response. They function as proficient antigen-presenting cells capable of producing a spectrum of proinflammatory and anti-inflammatory cytokines. Furthermore, B cells can generate terminally differentiated antibody-secreting plasma cells [69]. In respiratory diseases, the infiltration of B cells and production of immunoglobulins (such as IgM, IgG, and IgE) are correlated with disease severity [70]. Extensive studies have shown that MSCs inhibit the proliferation and differentiation of B cells into plasma cells and the production of immunoglobulins (IgM and IgG) [71–73]. CCL3 and CCL4 promote local inflow of neutrophils in the body, while some reports suggest that B cells express and secrete CCL3/4 [74]. Feng et al. found that MSC treatment inhibited the expression of the lung B-cell chemokine CCL4 in ALI mice, thereby reducing the entry of neutrophils into the injured lung tissue. In addition, the expression of the immunoglobulin-related genes *Iglc2*, *Iglc3*, and *Ighd* was reduced [75]. HAMSCs also influence the dynamics of B-cell recruitment and homing in bleomycin-induced PF by inhibiting the chemokine CXCL13 [21]. CpG ODN-2006, a synthetic oligonucleotide, induces B-cell proliferation and differentiation [76]. Parolini et al. explored the mechanism of action of hAMSCs on B cells. They found that hAMSCs could influence early CpG-induced B cell stimulation by reducing the expression of three major CpG sensors (CD205, TLR9, and CD14), thereby inhibiting downstream inflammatory signaling pathways [77] (Table 2, Fig. 2).

## Challenges for MSC therapy in lung diseases

Although the transplantation of MSCs has the potential to relieve lung diseases, the largescale application of MSCs in clinical settings still faces great challenges. Currently, the clinical applications of MSCs in treating lung diseases are mainly focused on severe coronavirus disease 2019 and acute respiratory distress syndrome, all of which are in phase I/II, with no largescale phase III clinical trials conducted.

Firstly, MSCs are heterogeneous cell populations, and MSCs from different donors and tissues exhibit different characteristics [78]. Researchers have compared MSCs derived from the umbilical cord, amniotic membrane, and bone marrow and found that although the three expressed similar surface markers, they had certain differences in paracrine factors, immunomodulatory ability, and regenerative support ability [79]. Tai et al. demonstrated that MSCs derived from different types of placental tissues exhibit distinct biological characteristics. Furthermore, heterogeneity was observed among MSCs of the same type sourced from different individuals [80]. With an increase in donor age, the multi-lineage differentiation, homing, immune regulation, and oxidative stress regulation of MSCs gradually decline and disappear [81]. In addition, the in vitro inoculation density of MSCs, different components in the growth medium (serum and growth factor combination), and oxygen concentration may affect the gene profile, epigenomic status, and phenotype of the cells [82]. As the number of cell passages increases, the expression profile of MSCs also changes; therefore, the most commonly used therapeutic passages are three to seven [83]. The result of the heterogeneity is a complexity that leads to challenges in their identification. Because the MSC subpopulation is not clearly defined and is difficult to distinguish, different combinations of markers are needed to confirm MSCs of different origins. The most commonly used markers are CD29, CD44, CD73, CD90, and CD105, whereas among the negative markers, CD34, CD45, and HLA-DR are the most commonly used [84]. Therefore, ensuring the quality, purity, potency, and stability of MSCs is one of the urgent challenges.

Secondly, the safety of MSC transplantation in vivo is a major challenge for its clinical application, including concerns about immunocompatibility, tumorigenicity, and microvascular occlusion [85]. Currently, most administrations are intravenous, and the limited number of MSCs reaching the injury site, along with a low survival rate, have restricted their therapeutic effectiveness owing to the presence of the immune microenvironment [86]. Apoptosis or autophagy occurs rapidly in MSCs following systemic or intratracheal administration [87]. Appropriate pretreatment of the cell culture medium can potentially modify the properties and therapeutic efficacy of MSCs. Preconditioning includes strategies, such as transgenics, exposure to hypoxia, administration of inflammatory factors, use of bioactive compounds, cultivation in 3D cultures, co-culture with disease-associated cells, or supplementation with patient serum. Furthermore, the clinical translation of stem cells requires ethical and safety considerations. MSCs’ ability to self-renew may pose a risk of tumor formation after entering the body [88]. The pro-tumor effects of MSCs manifest through various mechanisms within the tumor microenvironment, including differentiation into stromal components of the tumor microenvironment, suppression of immune responses, promotion of angiogenesis, enhancement of tumor cell survival, and facilitation of metastasis [89]. Moreover, there is a risk of thrombus formation when MSCs are transfused in vivo. In a Phase I/IIa study using Wharton’s jelly-derived MSCs for compression fractures, one patient in the stem cell treatment group developed pulmonary embolism [90]. Compared to observations of MSCs, MSC-EVs have higher biocompatibility and do not show evidence of carcinogenic capacity or a negative immune response [91, 92]. Additionally, MSC-EVs are easier to store and exhibit extremely high stability. They also demonstrate good biobarrier penetration and reduce the risk of microvascular embolization [93]. However, the lack of largescale production and extraction technologies, recognized standardized EV separation and characterization technologies, and handling and storage technologies have limited the clinical application of MSC-EVs [94]. In addition, determining the optimal route of administration and therapeutic dose required for different diseases using both MSCs and MSC-EVs is challenging [95, 96]. These issues have limited the clinical application of MSCs, and further research is needed to overcome these challenges.

## Conclusions

Effective treatments for lung diseases are still lacking, and researchers are striving to find new and effective drugs. MSCs and their derived secretomes exhibit protective effects against lung diseases, suggesting a potential therapeutic approach. Immune cells play crucial roles in the progression of lung diseases. MSCs have immunomodulatory properties; they can act on neutrophils, macrophages, T cells, and B cells; and play a role in both innate and adaptive immune responses during lung diseases. However, MSCs and their secretomes face challenges in clinical applications, such as heterogeneity, unsafe transformation in vivo, low survival rate, and lack of standardized methods for the isolation, extraction, and storage of EVs. In the future, more comprehensive basic research and larger clinical trials are required to address these issues.

## Data Availability

The information supporting this study’s findings is available in this article.
